# Analysis and Optimization of Light Absorption and Scattering Properties of Metal Nanocages

**DOI:** 10.3390/nano14191603

**Published:** 2024-10-04

**Authors:** Enhao Shao, Paerhatijiang Tuersun, Dilishati Wumaier, Shuyuan Li, Aibibula Abudula

**Affiliations:** 1 Xinjiang Key Laboratory for Luminescence Minerals and Optical Functional Materials, School of Physics and Electronic Engineering, Xinjiang Normal University, Urumqi 830054, China; eshao2-c@my.cityu.edu.hk (E.S.); lishuyuan@xjnu.edu.cn (S.L.); 2 Department of Physics, City University of Hong Kong, Kowloon, Hong Kong, China; 3 School of General Education, Xinjiang University of Technology, Hetian 848011, China; 18099246034@163.com; 4 School of Medical Technology, Xinjiang Hetian College, Hetian 848011, China

**Keywords:** metal nanocages, localized surface plasmon resonance, light absorption, light scattering, finite-element method

## Abstract

Metal nanocages exhibit localized surface plasmon resonance that strongly absorbs and scatters light at specific wavelengths, making them potentially valuable for photothermal therapy and biological imaging applications. However, investigations on metal nanocages are still confined to high-cost and small-scale synthesis. The comprehensive analysis of optical properties and optimal size parameters of metal nanocages is rarely reported. This paper simulates the effects of materials (Ag, Au, and Cu), size parameters, refractive index of the surrounding medium, and orientation on the light absorption and scattering characteristics of the nanocages using the finite-element method and the size-dependent refractive-index model for metal nanoparticles. The results show that the Ag nanocages have excellent light absorption and scattering characteristics and respond significantly to the size parameters, while the refractive index and orientation of the surrounding medium have less effect on them. The Au nanocages also possess superior light absorption properties at specific incident wavelengths. This study also identified the optimized sizes of three metal nanocages at incident light wavelengths commonly used in biomedicine; it was also found that, under deep therapy conditions, Ag nanocages in particular exhibit the highest volume absorption and scattering coefficients of 0.708 nm^−1^ and 0.583 nm^−1^, respectively. These findings offer theoretical insights into preparing target nanocage particles for applications in photothermal therapy and biological imaging.

## 1. Introduction

Cancer, one of the incurable diseases plaguing humankind today, has caused millions of deaths [1,2]. With the development of science and technology, many cancer diagnostic and therapeutic solutions have been developed, among which photothermal therapy (PTT) and bio-imaging are emerging cancer treatment technologies [3]. PTT offers fewer side effects compared to traditional surgical cutting, chemotherapy, and radiotherapy [2,4,5,6]. By utilizing the enhanced penetration and retention effect of solid tumors, PTT employs a photothermal agent (PTA) that accumulates in the tumor tissue. When irradiated with a near-infrared (NIR) laser, the PTA experiences photothermal conversion, raising the temperature to the point of destroying cancerous cells and curbing disease progression [7,8]. In the selection of PTA, a wide range of nanoparticles, both magnetic and nonmagnetic nanoparticles, can be effectively used in PTT [6]. Bio-imaging, a key technology in modern medical diagnostics, offers higher resolution and sensitivity than traditional tissue sectioning, radiography, and ultra-sound imaging [9]. Bio-imaging makes use of the special photophysical properties of the contrast agent (CA) to correlate the imaging signal with tumor cells in vivo; to present information about the structure, function, and metabolic process of the organism in the form of an image; and to realize the early detection, localization, and diagnosis of the disease [8,10,11]. CA is central to imaging research, particularly nanoparticle-based multifunctional CA, which has garnered significant attention in current research [12,13]. Metal nanoparticles are currently ideal photothermal and contrast agents in cancer diagnostic and therapeutic studies.

The application of metal nanoparticles in PTT and bio-imaging is mainly due to their localized surface plasmon resonance (LSPR), which exhibits strong light absorption and scattering characteristics [7]. In the current preparation and experimental study of metal nanoparticles, the LSPR properties are mainly tuned by changing the materials, size, shape, and other parameters of the metal nanoparticles [14,15,16,17,18]. The preparation of nanoparticles of conventional shapes, such as nanorods, ellipsoids, rods, and shells, is well established and their LSPR properties have been comprehensively studied [7,19,20,21,22]. Metal nanocages (NCs) are a novel type of nanoparticle characterized by hollow structures and mesoporous surfaces [23]. They offer higher sensitivity and tunability compared to conventionally shaped particles, along with a larger surface area that provides additional active sites. The synthesis of metal NCs has advanced significantly in recent years, mainly through the synthesis of various NCs by controlled etchant (e.g., HAuCl_4_), such as volume-porous nanoboxes, surface-porous NCs, and nanoframes [16,24,25,26,27,28,29,30]. Chen et al. prepared Au NC structures with large light absorption and scattering cross sections. Their light absorption and scattering properties can be tuned by controlling their dimensions, as well as the thickness and porosity of their walls [31]. Yavuz et al. prepared a metal NC-covered polymer for light-controlled release in the NIR region, which has strong light absorption properties for photothermal effects [32]. Gao et al. explored a morphologically stable method for the preparation of NCs with high activity, tunable dissociative excitatory activity, and adjustable size [33]. However, previous studies focused mainly on experimentally prepared irregular particles, with less emphasis on ideal NC parameters. Therefore, this paper simulates the light absorption and scattering characteristics of different metal NCs, aiming to provide theoretical insights for the experimental preparation of NCs with desired properties.

Commonly used methods for simulating the optical properties of non-spherical nanoparticles include the finite-element method (FEM), finite-difference time-domain (FDTD), discrete dipole approximation (DDA), and the boundary element method (BEM) [7,34,35]. Among these methods, FEM stands out for its flexibility and versatility, allowing for the analysis of complex geometries, material properties, and multiple types of interacting complex physical fields [36]. FEM discretizes the solution area of the electromagnetic field into small cells and numerically computes the electromagnetic field distribution of each cell [37,38]. In this study, we employ FEM and a size-dependent refractive-index model to simulate the light absorption and scattering characteristics of Ag, Au, and Cu NCs. We optimize the size parameters of NCs to achieve maximum values of light absorption and scattering.

## 2. Model and Method

### 2.1. Simulation Model

**Figure 1:**
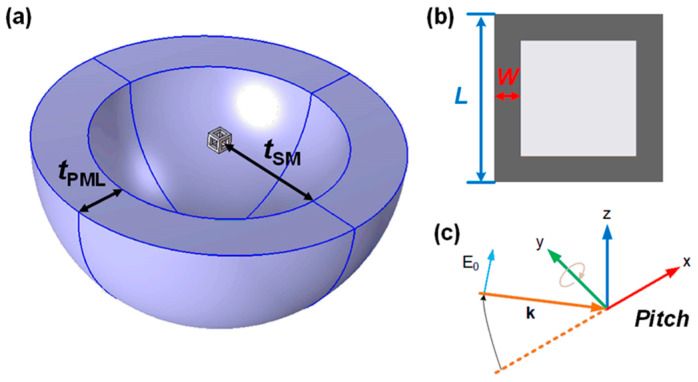
(**a**) FEM simulation region of NC; (**b**) Dimensional parameters of NC; (**c**) Schematic of three-dimensional coordinates (consistent with the orientation of (**a**)).

The type of metal NC studied in this paper is a hollow square with eight open sides (Figure 1a), whose geometry can be determined by *L* for the edge length and *W* for the particle wall thickness (Figure 1b). Due to the hollow architecture of NCs, the light absorption and scattering will be slightly changed under different angles of incident light. This effect can be analyzed by changing the angle of inclination (*Pitch*) of the incident light, which is a right-handed rotation with respect to the +y direction. as shown in Figure 1c [39]. The initial incident light propagates to the +x direction (*Pitch*: 0 rad, orange dashed line), and the electric field component of the incident light oscillates to the +z direction, corresponding to the +k and +E_0_ directions, respectively, after the angle change. The rotation axis of the NC is in the xoz plane, and *Pitch* denotes the angle between the rotation axis and the x-axis [7]. The FEM simulation region of the NC can be divided into the particle layer, the surrounding medium (SM) layer, and the perfect matching layer (PML), as shown in Figure 1a. The SM layer defines the calculation range of the light absorption and scattering parameters, where the parameters include the refractive index of the medium (biological tissue: 1.30–1.55), absorption cross section, scattering cross section, etc. The outer boundary of the SM layer is the scattering boundary, and the thickness of it (*t*_SM_) is set to be *λ*/2. The PML is a nonphysical receiver created outside of the SM layer, whose wave impedance is independent of the angle of incidence as well as the frequency of the scattering angle [40]. The PML acts as a truncation of the computational region in the numerical method to simulate problems with open boundaries; the wave is completely absorbed in the PML, whose thickness *t*_PML_ is set to *λ*/4. In addition to considering the two parameters—*L* and *W* of the particle size—the ratio of the two, *L*/*W*, can be taken as a parameter of the size, which is numerically equivalent to the aspect ratio of a prismatic cross section of the NC (aspect ratio, *AR* = *L*/*W*). Therefore, the dimensional parameters of an NC can be regulated by changing the *AR* during further simulation.

### 2.2. Fundamental Theory

The light absorption and scattering capacities of individual nanoparticles can be quantitatively described by the absorption cross section *σ*_abs_ and scattering cross section *σ*_sca_, which is defined as the ratio of optical power absorbed and scattered by the nanoparticle to the incident light intensity, respectively [41,42]:

(1) $$\sigma_{\mathrm{abs}}=\frac{1}{I_{i}}{∭}_{V_{P}}Q_{h}dV$$

(2) $$\sigma_{\mathrm{sca}}=\frac{1}{I_{i}}{∯}_{S}{\vec{P}}_{\mathrm{oav}}\cdot \vec{n}\to \;\mathrm{dS}$$

where *Q*_h_ is the heat loss density, *V*_p_ is the volume of the particle, ${\vec{P}}_{\mathrm{oav}}$ is the time-averaged Poynting vector component of the scattered field, and $\vec{n}$ is the unit vector in the direction normal to the sphere. $I_{i}=\left(n_{m}E_{0}^{2}\right)/\left(2Z_{0}\right)$ is the magnitude of the incident Poynting vector, *n*_m_ is the refractive index of the surrounding medium, *E*_0_ is the amplitude of the incident electric field (*E*_0_ = 1 V/m in the simulation), and *Z*_0_ is the wave impedance of the vacuum.

Different sizes of nanoparticles contain different amounts of matter, which leads to different light absorption and scattering capacities. Therefore, to fairly compare the light absorption and scattering characteristics of nanoparticles with different sizes, volumetric absorption coefficients, and scattering coefficients, which are the ratios of the absorption cross section and scattering cross section to the volume of the nanoparticles, respectively [7]. *A*_abs_ = *σ*_abs_/*V*_P_ (volumetric absorption coefficient) and *A*_sca_ = *σ*_sca_/*V*_P_ (volumetric scattering coefficient), which can be used to study the light absorption and scattering properties of nanoparticles. The refractive index of metal nanoparticles is related to nanoparticle size. Since their particle size is smaller than the mean free path of free electrons, the collision of free electrons with the particle surface is enhanced. Therefore, the size-dependent refractive index of metal nanoparticles can be expressed as follows [43]:

(3) $$n_{\mathrm{nano}\;}\left(\omega ,L_{\mathrm{eff}}\right)=\sqrt{n_{\mathrm{bulk}\;}^{2}(\omega )+\frac{\omega_{p}^{2}}{\omega^{2}+i\omega v_{f}/l_{\infty }}-\frac{\omega_{p}^{2}}{\omega^{2}+i\omega \left(v_{f}/l_{\infty }+Av_{f}/L_{\mathrm{eff}}\right)}}$$

where *ω* = (2*πc*)/*λ* is the angular frequency of incident light, *c* is the propagation speed of light in vacuum, *v*_f_ is the free electron Fermi velocity, *ω*_p_ is the plasma frequency, *A* is a dimensionless parameter (close to 1), $L_{\mathrm{eff}}=R_{\mathrm{eff}}=\sqrt[{3}]{3V/4\pi }$ is the effective mean free path of free electron, and *n*_bulk_ is the complex refractive index of the bulk metal material. In this paper, Ag, Au, and Cu NCs are used as the subjects, and the values of the mean free path $l_{\infty }$, Fermi velocity *v*_f_, and plasma frequency *ω*_p_ for the three materials are obtained from published papers [44,45,46], as shown in Table 1. In addition, the data related to complex refractive index were obtained from the data measured by Christy and Jonhson in 1972 [47].

### 2.3. Numerical Verification of FEM

**Figure 2:**
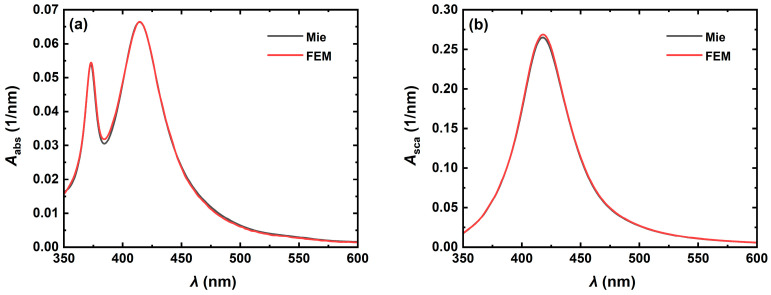
(**a**) Absorption and (**b**) scattering spectra of metal nanospheres with a radius of 30 nm in biological tissues with a refractive index *n*_m_ of 1.33.

To verify the accuracy and reliability of the FEM simulations, we compare the results of the FEM calculations with the rigorous Mie theory [48]. As shown in Figure 2, the absorption spectra (Figure 2a) and scattering spectra (Figure 2b) of Ag nanospheres with a radius *R* of 30 nm in biological tissues with a refractive index *n*_m_ of 1.33 are simulated using FEM and the Mie theory. The computational results show that the FEM simulation results are in good agreement with the strict Mie theory calculations, which fully verify the accuracy and reliability of the FEM simulation.

## 3. Results and Discussion

Based on the previous analyses, the light absorption and scattering properties of the NCs are mainly affected by material, size, surrounding medium, and orientation. To study the variation in light absorption and scattering of nanoparticles with special shapes according to various parameters, we selected preparable NCs as the research object and simulated the spectra of light absorption and scattering coefficients. In addition, the size parameters of the NCs were also optimized to make it suitable for PTT and bio-imaging. Considering that the wavelengths of incident light for PTT and bio-imaging applications are 808 nm, 1064 nm, and 800 nm, 980 nm, respectively, the wavelength range of incident light is taken to be from 700 nm to 1100 nm (the first NIR biological window).

### 3.1. Effect of Materials on Light Absorption and Scattering

**Figure 3:**
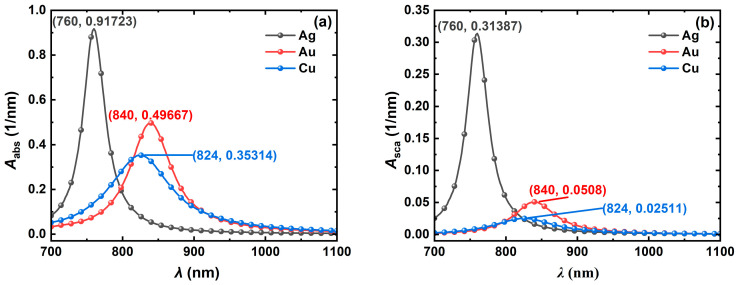
Variation in (**a**) volume absorption coefficient *A*_abs_ and (**b**) volume scattering coefficient *A*_sca_ with incident light wavelength *λ* for the Ag, Au, and Cu NCs. In the numerical simulation, the NC size *L* is 40 nm, the aspect ratio *AR* is 4, the refractive index *n*_m_ of the surrounding medium is 1.33, and the incident light inclination angle *Pitch* is 0°.

To compare the light absorption and scattering properties of different metal materials, the volume absorption and scattering coefficients of Ag, Au, and Cu NCs were simulated with wavelength as shown in Figure 3. The simulation results show that the Ag NC has large volume absorption and scattering coefficients of 0.917 nm^−1^ and 0.313 nm^−1^ at the resonance wavelength of 760 nm, which are 1.8 and 2.5 times higher than those of the Au and Cu NCs, respectively, and the volume scattering coefficients are 6.2 and 12.5 times higher than those of the Au and Cu NCs, respectively. The resonance wavelengths of the metal NCs are between 760 nm and 840 nm (Figure 3a,b), and the parameters can be further tuned to achieve optimal light absorption and scattering properties in the near-infrared region. In addition, the band in which free electrons resonate with incident light in the Ag NC is more concentrated, resulting in a narrower half-peak width of its spectrum, which can be better applied in biomedical fields. To tune the resonance wavelength of the NCs into the near-infrared band and to find the optimal parameters, the variations in the light absorption and scattering spectra of the Ag, Au, and Cu NCs concerning the dimensions, refractive index of the surrounding medium, and orientation will be further discussed. The light absorption and scattering properties of the metal NCs are mainly determined by the intensity and position of the resonance peaks, which are independent of the half-peak width, so only the trend of the resonance peaks is provided in the following sections.

### 3.2. Effect of Size on Light Absorption and Scattering

The dimensions of NCs can be regulated by edge length (L) and aspect ratio (AR). To investigate the variation rules of light absorption and scattering properties of the Ag, Au, and Cu NCs with their dimensions, this section quantitatively analyses the variation rules of the resonance peaks with the edge lengths and aspect ratios of the NCs.

#### 3.2.1. Effect of Edge Length

**Figure 4:**
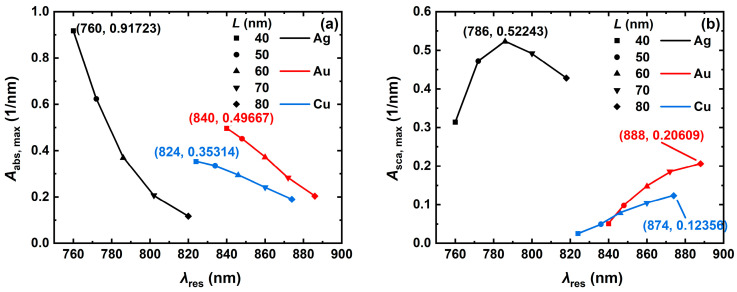
Trends of resonance peaks of (**a**) volume absorption coefficient and (**b**) volume scattering coefficient at resonance wavelength with edge length *L* for the Ag, Au, and Cu NCs. In the numerical simulation, the NC aspect ratio *AR* is 4, the refractive index *n*_m_ of the surrounding medium is 1.33, and the incident light inclination angle *Pitch* is 0°.

To quantitatively analyze the effect of edge length on the absorption and scattering spectra of the metal NCs, the trends of the resonance peaks of Ag, Au, and Cu NCs with various edge lengths *L* (40 nm, 50 nm, 60 nm, 70 nm and 80 nm) were simulated, as shown in Figure 4. The simulation results revealed that the absorption and scattering spectra of the Ag, Au, and Cu NCs are red-shifted as the NC edge length increases from 40 nm to 80 nm (Figure 4). The light absorption properties of the NCs of all the three materials are weakened at the resonance wavelength, with the largest change in the Ag NC, where the resonance wavelength is red-shifted from 760 nm to 820 nm, and the maximum volume absorption coefficient decreases from 0.917 nm^−1^ to 0.116 nm^−1^, as shown in Figure 4a. The Ag NC has a stronger light scattering property at the resonance wavelength compared to the Au and Cu NCs and has a stronger light scattering property at the resonance. The Ag NC has stronger light scattering than the Au and Cu NCs at the resonance wavelength and has the largest volume scattering coefficient of 0.522 nm^−1^ at the resonance wavelength of 786 nm, as shown in Figure 4b. When the edge length ranges from 40 nm to 60 nm, the volume absorption coefficient of the Ag NC at the resonance wavelength is significantly better than that of the same-size Au and Cu NCs. Its absorption responds significantly to the edge length, while the absorption of the Ag NC with the larger edge length is lower than the one of the Ag NC. The light scattering of the Ag NC is excellent at an edge length of 60 nm, and its light scattering is significantly better than that of the Au and Cu NCs in all nanoscale size ranges. With the increase in the edge length of NCs, its optical properties and electronic structure will be changed, which affects the absorption and scattering ability of light [49].

#### 3.2.2. Effect of Aspect Ratio

**Figure 5:**
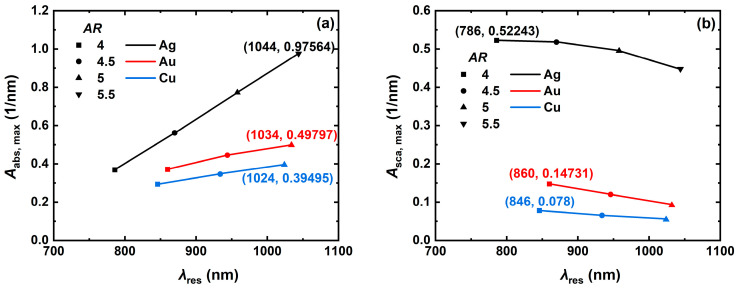
Trend of resonance peaks of (**a**) volume absorption coefficient and (**b**) volume scattering coefficient at resonance wavelength with aspect ratio *AR* for the Ag, Au, and Cu NCs. In the numerical simulation, the NC edge length *L* is 60, the refractive index *n*_m_ of the surrounding medium is 1.33, and the incident light inclination angle *Pitch* is 0°.

To quantitatively analyze the effect of aspect ratio on the absorption and scattering spectra of the metal NCs, the trends of the resonance peaks of the Ag, Au, and Cu NCs with aspect ratio *AR* (4, 4.5, 5, 5.5 and 6) were simulated as shown in Figure 5. The analysis results revealed that the resonance peaks of NCs with larger aspect ratios are red-shifted beyond the first NIR window (NIR-I: 700 nm to 1100 nm). To satisfy the optical bands usually used in the biomedical field, only the resonance wavelengths of the Ag, Au, and Cu NCs were simulated for the resonance peaks of the Ag, Au, and Cu NCs with the trend of resonance peaks with the *AR* within the NIR-I. The simulation results show that the tunable range of *AR* is about 4-5.5 for the Ag NC, while the tunable range of *AR* for the Au and Cu NCs should not be more than 5.5 in the NIR-I wavelength band. The absorption and scattering spectra of the Ag, Au, and Cu NCs show the same trend of changes with increasing *AR*, with the red-shifted position of resonance peaks and enhanced optical absorption characteristics at the resonant wavelength, and the light scattering property at the resonance wavelength is weakened. The most obvious changes were observed for the Ag NC, where the resonance peak position was red-shifted from 786 nm to 1044 nm, the maximum volume absorption coefficient was increased from 0.369 nm^−1^ to 0.976 nm^−1^ (Figure 5a), and the maximum volume scattering coefficient was decreased from 0.522 nm^−1^ to 0.448 nm^−1^ (Figure 5b). The absorption characteristics of the Ag NC responded better to *AR* and its resonance was higher than 4 at the *AR* of 0.522 nm^−1^. Its volume absorption coefficient at the resonance wavelength is significantly better than that of the Au and Cu NCs with the same *AR*, but the tunability of *AR* for the scattering properties of the NCs made of all the three materials is limited.

### 3.3. Effect of Surrounding Medium on Light Absorption and Scattering

**Figure 6:**
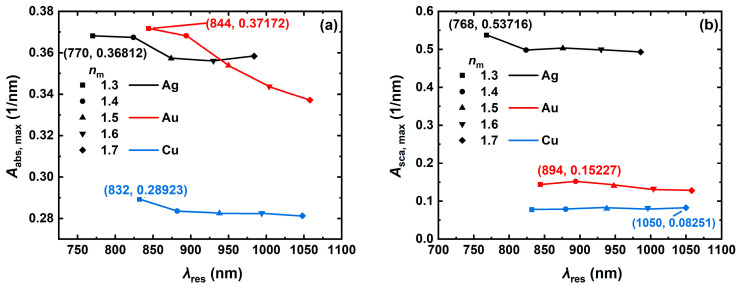
Trends of resonance peaks of light absorption (**a**) and scattering (**b**) with refractive index *n*_m_ of the surrounding medium for the Ag, Au, and Cu NCs. In the numerical simulation, the NC edge length *L* is 60, the aspect ratio *AR* is 4, and the incident light inclination angle *Pitch* is 0°.

To deeply analyze the effect of the surrounding environment on the light absorption and scattering properties of the metal NCs, the changes in volume absorption and scattering coefficients of the Ag, Au, and Cu NCs at resonance wavelengths in different surrounding media (refractive indices *n*_m_ of 1.3, 1.4, 1.5, 1.6 and 1.7, respectively) were simulated (Figure 6), which contains the refractive indices of biological tissues (usually in the range of 1.30–1.55). The refractive indices of biological tissues are similar to the refractive index of water. The simulation results show that as the refractive index of the surrounding medium of the NCs increases from 1.3 to 1.7, the absorption and scattering spectra of the Ag, Au, and Cu NCs are red-shifted with the increase in the refractive index of the surrounding medium, and the volume absorption and scattering coefficients at the resonance wavelengths fluctuate to different degrees. The largest change is the decrease in the volume absorption coefficient of the Au NC from 0.372 to 0.337 nm^−1^, as shown in Figure 6a. The decrease in the volume scattering coefficients of all the three metal NCs is less than 0.05 nm^−1^, as shown in Figure 6b. Unlike the other parameters, both the Ag and Au NCs have their excellent optical absorption properties at a particular range of ambient refractive indices. Overall, the refractive index of the surrounding medium has less influence on the optical absorption and scattering characteristics of the NCs. If Au is used as a research object, changing the refractive index of the surrounding environment can make it produce superior optical absorption characteristics. It is found that the resonance wavelengths of the three materials are significantly red-shifted with the increase in the NC edge length, aspect ratio, and ambient refractive index, which is attributed to the decrease in the intrinsic oscillation frequency of the free electrons in the nanoparticles, thus leading to the decrease in the resonance frequency and the increase in the resonance wavelength [50].

### 3.4. Effect of Incident Light Orientation on Light Absorption and Scattering

**Figure 7:**
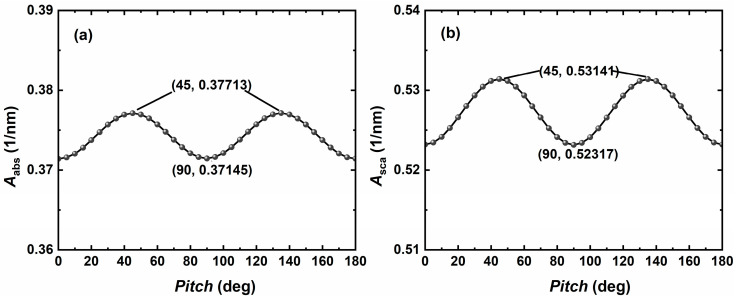
Variation in volume absorption coefficient Aabs (**a**) and scattering coefficient Aabs (**b**) with the incident light inclination for the Ag NC at 786 nm with an edge length *L* of 60, aspect ratio *AR* of 4, and refractive index nm of the surrounding medium of 1.33.

The orientation of metal nanoparticles is random, and the effects observed in clinical trials follow a statistical pattern. Since some metal nanoparticles are magnetic, they will move or rotate due to the magnetic field. To determine the high efficiency of metal NC particle populations in applications, the differences in light absorption and scattering properties of metal NCs in various orientations need to be verified. The orientation of the nanoparticles can be accurately modulated by changing the *Roll*, *Pitch*, and *Yaw* of the incident light. In this paper, the NC type is a hollow square, and the initial position is the positive direction of the incident light into one side of the metal NCs. Therefore, changing the *Roll* does not affect the optical properties produced by the metal NCs. In this study, we take changing the *Pitch* as an example and simulate and analyze the change rule of the light absorption properties of the metal NCs when the *Pitch* is rotated from 0° to 180°, as shown in Figure 7a,b. It is found that the light absorption characteristics of the metal NCs will produce a periodic change, and the magnitude of the change is 0.006 nm^−1^. Therefore, changing the azimuthal angle does not have a significant effect on the light absorption and scattering properties of the metal NCs.

### 3.5. Optimization of Light Absorption and Scattering

In cancer treatment, different wavelengths of incident light have different levels of penetration into biological tissues, and the right wavelength of incident light can reduce damage to normal cells and achieve a better therapeutic effect. Tumors can be treated superficially (skin cancer and neck enlargement) and deeply (liver, stomach, and esophagus). Superficial therapies use wavelengths of 800 nm (PTT) and 808 nm (bio-imaging), which are less damaging to normal cells, while deep therapies use wavelengths of 980 nm (PTT) and 1064 (bio-imaging), which have good penetration into biological tissue [51,52]. To meet the therapeutic needs of different types of tumors, wavelengths of 800 nm, 808 nm, 980 nm, and 1064 nm were selected as the light sources, and the optimal edge lengths and aspect ratios were calculated to obtain the maximum volume absorption and scattering coefficients.

#### 3.5.1. Optimum Size Parameters for PTT

**Figure 8:**
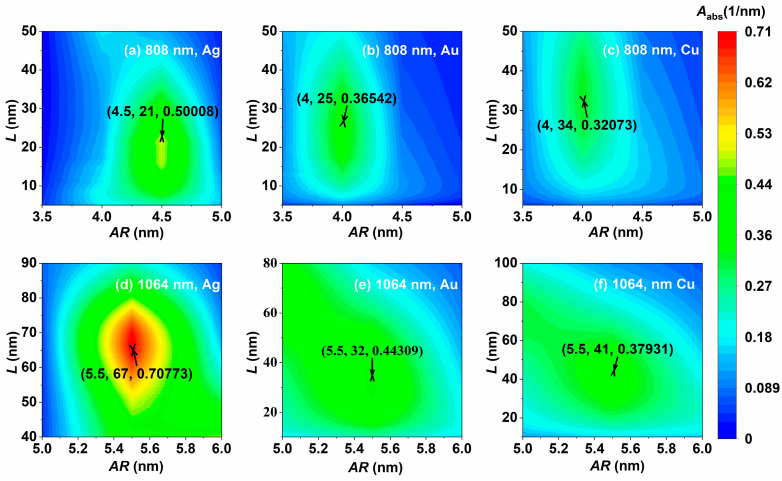
Optimization of light absorption of the Ag, Au, and Cu NCs at laser wavelengths of (**a**–**c**) 808 nm and (**d**–**f**) 1064 nm.

The incident light wavelengths applied to PTT are 808 nm (superficial therapy) and 1064 nm (deep therapy), and the simulation results are shown in Figure 8. When the incident light wavelength is 808 nm, the optimal aspect ratios of the NCs made of the three materials are similar, and the light absorption of the smaller Ag NC is significantly better than that of the Au and Cu NCs, as shown in Figure 8a–c. When the incident light wavelength is 1064 nm, the edge lengths, aspect ratios, and volume absorption coefficients of the three materials are significantly increased, as shown in Figure 8d–f. Whether the wavelength of incident light is 808 nm or 1064 nm, the Ag NC exhibits excellent light absorption properties; the Ag NC with an edge length of 67 nm and aspect ratio of 5.5 has the largest volume absorption coefficient at 0.708 nm^−1^ at 1064 nm incident light, as shown in Figure 8d. The optimal aspect ratios of the three materials are less affected by the materials and are mainly regulated by the incident wavelengths. The optimal aspect ratios at the same incident wavelengths are similar. It can be seen that the optical absorption of the Ag NC is superior to that of the Au and Cu NCs under the optimal size parameter. In addition, the hollow structure of the NCs allows multiple reflections of light inside the cage, which enhances the absorption. By comparing the volumetric absorption coefficients of the optimal Ag@TiO_2_ nanospheroid and nanorod simulated by Wumaier et al. [7], it can be found that the light absorption properties of optimally sized nanocages are superior to those of the Ag@TiO_2_ nanospheroid and nanorod.

#### 3.5.2. Optimum Size Parameters for Bio-Imaging

**Figure 9:**
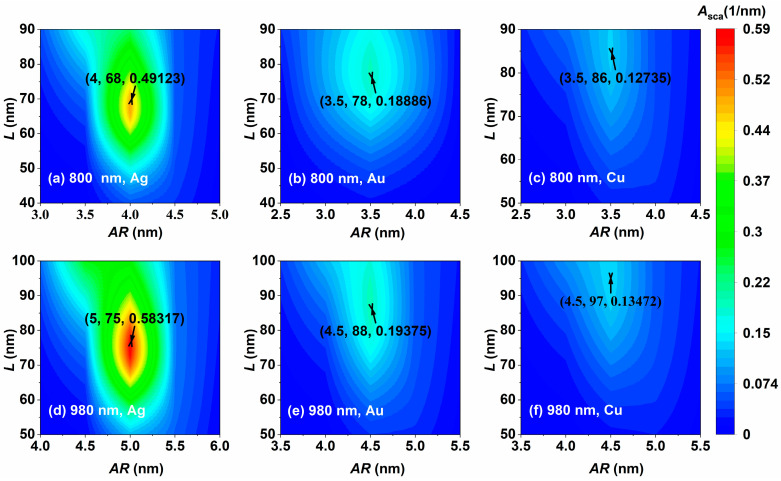
Optimization of light scattering of the Ag, Au, and Cu NCs at laser wavelengths of (**a**–**c**) 800 nm and (**d**–**f**) 980 nm.

The incident light wavelengths applied to bio-imaging are 800 nm (superficial therapy) and 980 nm (deep therapy), and the simulation results are shown in Figure 9. When the incident light wavelength is 800 nm, the optimal aspect ratios of the three materials NCs are similar, and all of them have larger optimal sizes, among which the light scattering capability of the Ag NC is significantly better than that of the Au and Cu NCs, as shown in Figure 9a–c. When the incident light wavelength is 980 nm, the edge lengths, aspect ratios, and volume scattering coefficients of the three materials are increased, as shown in Figure 9d–f. Whether the wavelength of incident light is 800 nm or 980 nm, the Ag NC shows excellent light scattering properties; the Ag NC with an edge length of 75 nm and aspect ratio of 5 has the largest volume scattering coefficient of 0.583 nm^−1^ under the 980 nm incident light, as shown in Figure 9d.The optimal aspect ratios of the different materials have a diversity of less than 0.5, and only by changing the wavelength of the incident light, would the aspect ratios have a larger degree of response. It can be seen that the light scattering characteristics of the Ag NC are superior under the optimum size parameters compared to the Au and Cu NCs.

In comparison, the light absorption and scattering properties of the Ag NC are superior at all classical wavelengths of incident light, and the optimized Ag NC can be used as an ideal photothermal and contrast agent. Despite the superior light absorption and scattering properties of the deep tumor therapy option, it should be used with sound judgment in actual therapy.

## 4. Conclusions

This paper investigates the variations in light absorption and scattering properties of Ag, Au, and Cu NCs with material, size parameters, ambient refractive index, and particle orientation. We also obtained the optimal edge lengths and aspect ratios of the three metal NCs at the incident wavelengths commonly used in PTT (808 nm and 1064 nm) and biological imaging (800 nm and 980 nm), respectively. The simulation results show that the Ag NC has better light absorption and scattering properties than the Au and Cu NCs, and its narrower half-peak width makes it better for biomedical applications. The dimensional parameters (edge length and aspect ratio) of the three metal NCs are obviously tunable, which can effectively change the intensity and position of the resonance peaks in the absorption and scattering spectra. Au NCs of a specific ambient species will be more effective for a specific incident light wavelength than those of the three metal NCs. Specific ambient species of Au NCs produce superior light absorption properties compared to Ag NCs at specific incident wavelengths, but the refractive index of the surrounding medium has little effect on the light absorption and scattering of the three metal NCs. The orientation of the metal NCs does not lead to changes in the resonance wavelength due to their hollow structure. The change in the orientation only causes a weak shift of the resonance peaks in the absorption and scattering spectra. Based on the simulation results of the optimal size parameters, it can be found that the optimal aspect ratios of the Ag, Au, and Cu NCs are less affected by their material and they are regulated mainly by the wavelength of the incident light. In the field of PTT and bio-imaging, the optical absorption and scattering capability of Ag NCs is optimal in deep therapy. These three materials require an additional layer of antioxidants on the surface of the nanoparticles due to their poor stability in avoiding their oxidization of the resulting from direct contact with oxygen. This is particularly crucial for Ag, which is toxic and requires encapsulation with non-toxic materials for biomedical applications. The four edges of the NC model can be further smoothed to better fit FEM analyses, which would allow one to obtain more accurate simulation data. This investigation provides theoretical references for biomedical, solar cell, and photocatalytic fields.

## Figures and Tables

**Table 1:** The values of Fermi velocity *v*_f_, mean free path of the free electrons *l*_∞_, and plasma frequency *ω*_p_ for Au, Ag, and Cu [44,45,46].

Metal	*v*_f_ (m/s)	*l*_∞_ (m/s)	ℏ*ω*_p_ (eV)
Au	1.40 × 10^6^	42	9.03
Ag	1.39 × 10^6^	52	9.01
Cu	1.57 × 10^6^	45	10.83

## Data Availability

The original contributions presented in this study are included in this article; further inquiries can be directed to the corresponding authors.
